# Editorial: Non-immunological care of the kidney transplant recipients

**DOI:** 10.3389/fneph.2024.1440359

**Published:** 2024-07-12

**Authors:** Matthieu Halfon, Olivier Bonny, Daniel Teta

**Affiliations:** ^1^ Transplantation Center, Lausanne University Hospital, Lausanne, Switzerland; ^2^ Service of Nephrology, Department of Medicine, State Hospital Fribourg and University of Fribourg, Fribourg, Switzerland; ^3^ Faculty of Biology and Medicine, Lausanne University, Lausanne, Switzerland; ^4^ Service of Nephrology, Valais Hospital, Sion, Switzerland

**Keywords:** transplantation, pregnancy, rejection, dementia, transfusion, nonimmunological, kidney, graft

The widespread incorporation of medications such as tacrolimus and mycophenolate, coupled with vigilant monitoring, along with advancements in anti-human leukocyte antibody testing and screening procedures, has played a crucial role in the substantial reduction of graft loss risk due to acute rejection over the past decade, and has contributed to the overall improvement in graft survival. In a study published in 2012, Sellare et al. highlighted graft rejection as the primary factor leading to graft loss (1). However, a decade later, a significant shift occurred, and graft rejection was replaced by chronic allograft nephropathy or “death with a functioning graft” as the main cause of graft loss (2, 3). Nonetheless, transplant nephrologists must remain vigilant as the specter of rejection looms constantly. Indeed, they navigate a delicate balance, grappling with the dual challenges of over-immunosuppression and its associated risks, such as heightened susceptibility to infection and increased risk of cancer,now recognized as major contributors to mortality in transplant recipients (4, 5). On the other hand, under-immunosuppression poses its own set of dramatic consequences with the risk of alloimmunization and subsequent graft rejection.

Due to the increased graft survival and the fact that more polymorbid patients have access to kidney transplantation, transplant nephrologists are increasingly assuming a role akin to that of primary care practitioners in their daily clinical care (6). They now encounter novel challenges that were seldom observed previously, such as managing pregnancies or addressing dementia in kidney transplant recipients. Additionally, they grapple with the long-term consequences of immunosuppressive drugs. This multifaceted responsibility demands a comprehensive approach to patient care that goes beyond the traditional scope of transplant medicine. In this special issue of Frontiers in Nephrology, we aim to spotlight critical facets of these “non-immunological aspects”. We intend to delve into the challenges that transplant nephrologists are increasingly encountering in their clinical practice, providing insights into the complexities they are dealing with.

In their investigation on blood transfusions, Hassan et al. highlighted that certain “non-immunological aspects” impact transplant patients from the early post-transplantation stage. Their comprehensive analysis of a large multicenter cohort revealed that approximately 30% of transplant recipients received blood transfusions very soon after the procedure (median time of 4 days post-transplant). Alarmingly, these transfusions were linked to adverse outcomes, as these patients exhibited a sixfold increase in the risk of mortality within one year and a threefold increase in the risk of graft loss compared to non-transfused patients. Notably, transfused patients were more likely to have received an organ from a donor after cardiac death or from a donor with expanded criteria. They also benefited less from pre-emptive kidney transplantation and had longer dialysis vintages, all of which partially accounted for the differences in survival and graft outcomes. However, even after considering these contributing factors, a significant difference in survival persisted, suggesting an inherent vulnerability in some patients. The work of Hassan et al. underscores the critical need for meticulous pre-operative management, such as optimizing pre-transplantation hemoglobin levels to decrease the risk of post-operative anemia and thereby reduce the need for blood transfusions. Moreover, it highlights the importance of attentive care in cases where transfusions are deemed necessary. The authors suggested that optimal use of erythropoietin-stimulating agent (ESA) therapy or iron therapy could help achieve these goals. However, it should be noted that the use of ESA therapy has been linked to an increased risk of cardiovascular events when specific hemoglobin or hematocrit thresholds are exceeded, and iron therapy may increase the risk of infection (7, 8). Future studies are needed to assess the efficacy, optimal pre-transplant hemoglobin targets, and safety of these therapies. Limitations of the study include the lack of data detailing the reasons for blood transfusion and the lack of information on hemoglobin levels at the time of transfusion. It is worth noting that transfusion practices may vary between centers, whether based on predefined thresholds or symptomatic indications, potentially impacting the study outcomes. Furthermore, the study’s inability to collect data on *de novo* alloimmunization during rejection episodes makes it difficult to draw definitive conclusions regarding the immunological risks associated with blood transfusions.

In their original research article, Stavart et al. addressed the complexities surrounding pregnancy in kidney graft recipients. Despite observing no graft loss among the 14 pregnancies studied over a two-year post-pregnancy follow-up period, and no discernible increase in alloimmunization, their data revealed certain noteworthy trends. The authors observed a decrease in glomerular filtration rates between pre-conception and post-delivery phases, with 30% of patients displaying an additional increase in proteinuria. Importantly, nearly half of the women underwent a kidney biopsy due to elevated creatinine and proteinuria levels after pregnancy. Results included calcineurin inhibitor toxicity in three patients and acute T cell-mediated rejection in two patients. The study also provides valuable insight into the outcomes of the offspring. Although all pregnancies resulted in successful births, the majority of infants were premature, with 30% of these premature births being induced, and they displayed low birth weight. However, the authors did not provide data regarding the causes of premature birth or low birth weight. This study underscores the critical need for planned pregnancies and emphasizes the need for consistent monitoring and care throughout the gestational period for these high-risk pregnancies. However, the study has certain limitations, such as the overall short follow-up period after pregnancy and the lack of a low-dose aspirin prescription in the majority of patients. It is noteworthy that low-dose aspirin has been shown to significantly reduce the risk of preeclampsia, especially in high-risk patients (9). Following the publication by Rolknik et al., the use of aspirin in kidney transplant recipients has become a common practice in transplantation centers, even in the absence of specific recommendations for kidney transplant recipients (10). It is important to note that the favorable outcomes observed in the mothers may have been influenced by the absence of patients suffering from conditions prone to flare during pregnancy, such as systemic lupus erythematosus. Furthermore, the inclusion criteria, which focused solely on patients with live births may inadvertently have missed women who exclusively experienced pregnancies with unfavorable outcomes. Finally, despite providing information on eGFR during follow-up, the authors did not include data on the evolution of proteinuria.

Polychronopoulou et al. discussed the use of SGLT2 inhibitors (SGTL2i) in kidney transplant recipients (KTx) in their review. They pointed out the potential positive impact of this class of drugs on KTx.

Kidney transplantation is characterized by glomerular hyperfiltration, which could potentially be alleviated by SGTL2i and contribute to nephroprotection to some extent (11). Additionally, kidney transplant recipients are considered a high cardiovascular risk group and could also benefit from the cardioprotective effects of SGLT2i. However, Polychronopoulou et al. pointed out the current lack of robust studies in KTx, as the majority of studies are retrospective, have short follow-ups, and were not designed to specifically assess renal or cardioprotective effects. On the positive side, current studies have not shown an increased risk of urinary tract infections, but most of them have included patients transplanted for more than one year because of a higher incidence of urinary tract infections in the first year after kidney transplantation. As summarized by the authors, large prospective studies with longer follow-ups and designed to assess renal and cardiovascular outcomes are currently underway. We hope that these studies will help answer questions regarding the safety and efficacy of these drugs in both diabetic and non-diabetic KTx patients.

Finally, Schietzel et al. provided invaluable insights into cognitive impairment in kidney transplant patients. Despite initial improvements in cognition during the early post-transplant period, the prevalence of cognitive impairment in kidney transplant recipients is still high as it can range from 15 to 58% depending on the definition used. This prevalence indicates that virtually every transplant nephrologist will encounter this issue in their practice. Furthermore, the study highlights that cognitive impairment not only complicates the overall care of patients but also contributes to an elevated rate of mortality and graft loss. These findings underline the importance of addressing cognitive health as an integral part of pre - and post- transplant care, stressing the need for tailored interventions to effectively manage this aspect.

In conclusion, the evolving landscape of kidney transplantation demands a holistic approach in conjunction with the traditional focus on immunological factors. The intricate web of non-immunological aspects has emerged as a critical element of transplant care, adding a layer of complexity in the careful management of the risk of rejection and the consequences of long-term immunosuppression. As transplant nephrologists navigate this new paradigm, meticulous pre-operative management, vigilant post-transplant monitoring, and tailored interventions become paramount. Research elucidating these non-immunological dimensions underscores the imperative for comprehensive, patient-centered care that not only safeguards graft longevity but also prioritizes the overall well-being of transplant recipients. By recognizing and addressing these multifaceted aspects, we are paving the way for better outcomes, an improved patient experience, and a more comprehensive understanding of kidney transplantation in the realm of modern medicine.

## Author contributions

MH: Writing – original draft, Writing – review & editing. OB: Writing – original draft, Writing – review & editing. DT: Writing – original draft, Writing – review & editing.
